# Molecular Evolutionary Analysis of the HCRTR Gene Family in Vertebrates

**DOI:** 10.1155/2018/8120263

**Published:** 2018-06-04

**Authors:** Zhen Cai, Hehe Liu, Liyun Wang, Xinxin Li, Lili Bai, Xinmeng Gan, Liang Li, Chunchun Han

**Affiliations:** ^1^Farm Animal Genetic Resources Exploration and Innovation Key Laboratory of Sichuan Province, Sichuan Agricultural University, Chengdu, Sichuan 611130, China; ^2^The First Affiliated Hospital, Zhejiang University School of Medicine, Hangzhou 310003, China

## Abstract

Hypocretin system is composed of hypocretins (hcrts) and their receptors (hcrtrs), which has multiple vital functions. Hypocretins work via hypocretin receptors and it is reported that functional differentiation occurred in hcrtrs. It is necessary to figure out the evolution process of hypocretin receptors. In our study, we adopt a comprehensive approach and various bioinformatics tools to analyse the evolution process of HCRTR gene family. It turns out that the second round of whole genome duplication in early vertebrate ancestry and the independent round in fish ancestry may contribute to the diversity of HCRTR gene family. HCRTR1 of fishes and mammals are not the same receptor, which means that there are three members in the family. HCRTR2 is proved to be the most ancient one in HCRTR gene family. After duplication events, the structure of HCRTR1 diverged from HCRTR2 owing to relaxed selective pressure. Negative selection is the predominant evolutionary force acting on the HCRTR gene family but HCRTR1 of mammals is found to be subjected to positive selection. Our study gains insight into the molecular evolution process of HCRTR gene family, which contributes to the further study of the system.

## 1. Introduction

Hypocretin system is composed of hypocretins (hcrts) and their receptors (hcrtrs). In vertebrates, there are two hypocretins, hcrta and hcrtb. Both of them are derived by proteolytic processing from a common precursor preproorexin coded by HCRT [1]. Hypocretins work via hypocretin receptors. Up to now, there are two types of receptors found in vertebrates, named hcrtr1 and hcrtr2. hcrtr2 has similar affinity for both hypocretins, whereas hcrtr1 favors hcrta [2]. Since discovered in 1998 [3, 4], hypocretin system has been reported to participate in various physiological processes including dietary and metabolic regulation [5], sleep-wake circuit [6, 7], drug addiction and reward process [8], and even cancerous cells apoptosis [9–13]. Studies on knockout or neuron-depleted animal models indicate hypocretin system is important in maintaining normal vital signs such as basal blood pressure [14], respiratory rate [15], and energy expenditure [16–18].

Given the diverse functions of hypocretin system, antagonists targeted at hcrtr1 or hcrtr2 are considered as potential drugs for treatment of many diseases. Take insomnia, for example. Existing drugs such as benzodiazepines and melatonin receptor antagonists play a role in the treatment of insomnia, but most of them will lead to the change of sleep architecture, with many side effects. It has been found that hypocretin plays a crucial role in the sleep regulation and can be used as a novel therapeutic target for insomnia. Hypocretin receptor antagonists can selectively block hypocretin receptors with no side effects like benzodiazepines, and they have stronger pharmacological activity than existing hypnotics. This not only provides a new approach for the treatment of insomnia, but also provides an alternative treatment strategy for other hcrtr-mediated pathologies such as drug addiction, obesity, and depression. At present, the development of orexin receptor antagonists has made great progress. Suvorexant [19] has already been launched to the market. Lemborexant [20], Filorexant [21], and SB-649868 [22] are in phase II or phase III trials, and MK-1064 [23] and MK-3697 [24] have become clinical candidate drugs. There is huge potential and great prospect for the drug researches.

Moreover, functional differentiation occurred in hcrtrs. Dogs and mice lacking functional HCRTR2 are observed to show narcolepsy [25, 26], yet HCRTR1 knockout mice hardly act abnormally in the sleep-wake circuit [17]. Hcrtrs are synthesized solely within a restricted region of the dorsal hypothalamus, including the lateral hypothalamus proper, adjacent perifornical area, and dorsomedial hypothalamus. Despite that, hypocretin neurons project widely throughout the brain targeting two receptors with partially overlapping distributions [27]. Hcrtr2 in histaminergic neurons of tuberomammillary is a key mediator for stimulating arousal, and hcrtr2 in PVN and autonomic nuclei of medulla plays a role in stress responses. Hcrtr1 mainly exists in LC and areas in control of food intake and motivated behaviors [28].

With more and more genomes sequenced, an increasing number of HCRTR sequences spring up. HCRTR1 forms after mammals appear. There was a long time that two hypocretins corresponded to one receptor. It is interesting to figure out the evolution process of the hypocretin receptors. However, the evolution process of hypocretin system has rarely been discussed. Once, scientists thought that hypocretin and secretin were evolutionarily relevant because they shared similar sequence [3, 29]. However, the study between hypocretin/secretin and their receptors rejected the point [30]. Because HCRTR2 is widespread in vertebrates and HCRTR1 is specific to mammals, it is generally believed that HCRTR2 is more ancient and that HCRTR1 forms in mammals from HCRTR2 [31]. However, the HCRTR1 found in fishes indicates that HCRTR gene family is more complicated than anticipated. A thorough evolution analysis is yet to be conducted.

In our study, we adopted a comprehensive approach and various bioinformatics tools to analyse the evolution process of HCRTR gene family, which gains insight into hypocretin system. Tons of researches demonstrate that hypocretin system is closely related to a large amount of diseases, such as obesity [32, 33], panic anxiety [34], age-related anorexia [35], multiple system atrophy [36], neurological disorders [37], Parkinson's disease [38], Alzheimer disease [39], and obstructive apnea-hypopnea syndrome [40]. Different receptors may be linked to different diseases. There are crystal clear benefits to figure out the evolution process of HCRTR gene family.

## 2. Materials and Methods

### 2.1. Mining Databases and Collecting Sequences

The coding sequences and amino acid sequences of HCRTR gene family were downloaded from the public databases, NCBI (http://www.ncbi.nlm.nih.gov/) and Ensembl (http://asia.ensembl.org/index.html), which involved a total of 166 species (Additional File 1). 38 representative species were chosen (Additional File 1) and Blastn (https://blast.ncbi.nlm.nih.gov/Blast.cgi) was used to retrieve HCRTR-likes in respective genome with its own HCRTR1 sequence as reference. Most uncertain sequences were deleted after alignment.

### 2.2. Prediction of Protein Structure Domains (PSDs)

The amino acid sequences were submitted to SMART (http://smart.embl-heidelberg.de/) to predict protein structure domains. After PSDs were predicted, we obtained the corresponding nucleotide and amino acid sequences from the data set we collected above. Because the difference mainly consisted in one structure domain (Orexin_rec2), we then compared Orexin_rec2 domains of 11 representative species including human, mouse, cow, platypus, zebra finch, chicken, zebrafish, tiger puffer, West Indian Ocean coelacanth, American alligator, and African clawed frog.

### 2.3. Phylogenetic Analysis

The amino acid sequences of the conserved structure domain (7tm_1) were aligned with MAFFT [41] with default parameters. Before phylogenetic tree was constructed, the best model was predicted with ProtTest 3.4.2 [42]. Phylogenetic tree was calculated with 1000 bootstrap replicates with two methods, Maximum likelihood and Neighbor-joining, under JTT + G model. Vase tunicate was the out-group in both trees.

### 2.4. Gene Microsynteny Analysis

Gene microsynteny analysis was performed with a public platform Genomicus (http://www.genomicus.biologie.ens.fr/) on which we obtained microsynteny of HCRTR gene family. Owing to the limited species, we selected 12 species for further analysis including human, mouse, cow, chicken, zebra finch, coelacanth, zebrafish, spotted gar, fugu, frog, lizard, and vase tunicate.

### 2.5. Selective Pressure Analysis

We analysed all the conserved PSDs of hcrtrs, respectively. Because the sequence number of 7_tm1 structure domain of hcrtr1 of fishes was not enough for analysis and no Orexin_rec2 structure domain was predicted from the amino acid sequences of hcrtr1s, we just divided PSDs into three group: Group (1) 7_tm1 structure domain of hcrtr1 among mammals; Group (2) 7_tm1 structure domain of hcrtr2; Group (3) Orexin_rec2 structure domain of hcrtr2. Unreliable sequences were deleted after alignment in case of false annotation. The nucleotide sequences were aligned with Muscle (Codons) in MEGA7 [43]. We then analysed selective pressure of each group with Datamonkey (http://www.datamonkey.org/) [44] to detect positive selection sites. The automatic model selection tool was executed to choose the best model and three groups matched three different nucleotide substitution bias models. Also, two methods (SLAC and FEL) were used to analyse selective pressure of each site.

## 3. Results

### 3.1. Members of HCRTR Gene Family

We obtained a set of HCRTR sequences from 109 species which consisted of 48 HCRTR1s and 109 HCRTR2s (Additional File 1). In the past, it was universally believed that HCRTR1 was specific to mammals while HCRTR2 existed in all the vertebrates. However it turned out that there were three HCRTR1-likes in fishes, one named HCRTR1 in West Indian Ocean coelacanth (*Latimeria chalumnae*), one annotated HCRTR1 in spotted gar (*Lepisosteus oculatus*), and one without annotation sharing 48% common sequence with HCRTR2 of Atlantic salmon (*Salmo salar*). In addition, vase tunicate (*Ciona intestinalis*), a type of Urochordata, was found to have HCRTR2 as well. More and more exceptions indicated that HCRTR gene family was larger than anticipated.

During analysis, we also found a false annotation in NCBI database. Detailed phylogenetic and structure analysis suggested that the annotated HCRTR1 of Dalmatian pelican (*Pelecanus crispus*) was more similar to HCRTR2. There was no HCRTR1 in birds.

### 3.2. Phylogenetic Analysis of HCRTR Gene Family

**Figure 1:**
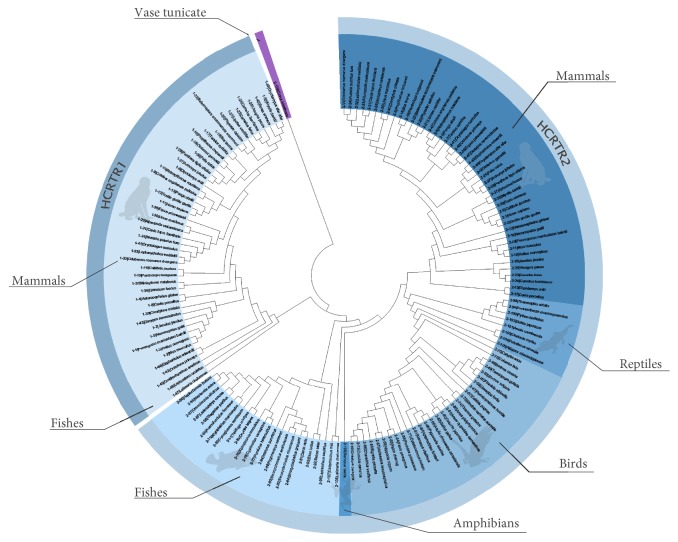
Phylogenetic tree (Neighbor-joining). All the information is indicated with different colors and annotations in the figure. The label of each branch is shown in the form of x-x|xxx. The first x indicates gene family the gene belongs to. The second x stands for its order in list of Additional File 1. And xxx represents the scientific name of species.

**Figure 2:**
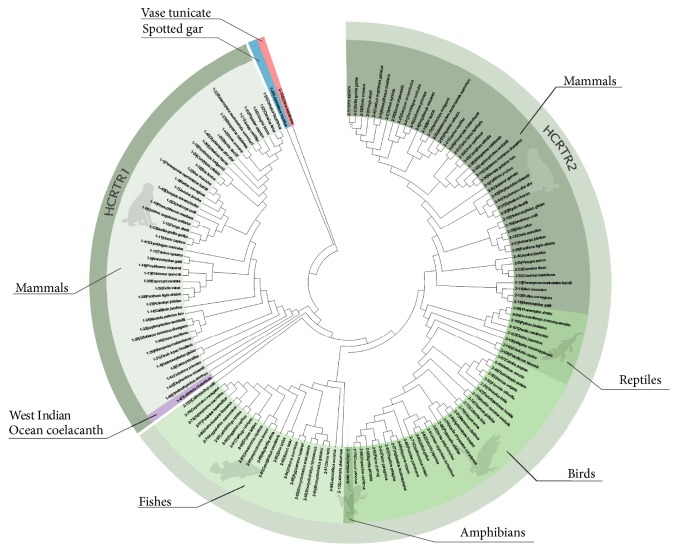
Phylogenetic tree (Maximum likelihood). All the information is indicated with different colors and annotations in the figure. The label of each branch is shown in the form of x-x|xxx. The first x indicates gene family the gene belongs to. The second x stands for its order in list of Additional File 1. And xxx represents the scientific name of species.

The phylogenetic trees with two methods were similar and highly consistent with the species tree (Figures 1 and 2). HCRTR1 and HCRTR2 diverged in a very early time in both trees and HCRTR2 of vase tunicate (out-group) was not grouped into any clade of vertebrates. However, in Neighbor-joining tree (Figure 1), HCRTR1s of spotted gar and West Indian Ocean coelacanth were grouped into HCRTR1 clade while in Maximum likelihood tree (Figure 2) HCRTR1 of spotted gar did not belong to any existing clades and HCRTR1 of West Indian Ocean coelacanth was grouped into HCRTR2 clade. In both trees, no matter what clade HCRTR1 of fishes was grouped into, they diverged from the common ancestors very early.

### 3.3. Gene Microsynteny Analysis

**Figure 3:**
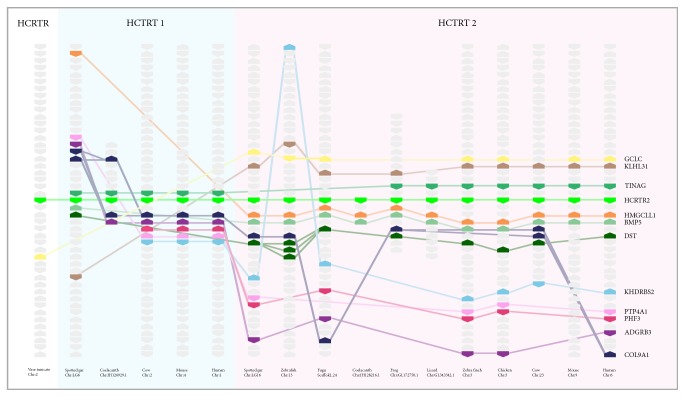
Gene microsynteny. Rectangles in different colors represent different flanking genes whose names are shown on the right side of the figure. Arrows illustrate the directions of genes. The same genes are linked by lines in the same colors. The species and the chromosomes where HCRTRs are located are shown below.

Genes around HCRTR gene family were conserved and arranged in a particular order (Figure 3). We showed 11 genes that were close to HCRTR gene family and it could be seen that genes around HCRTR2 were more conserved. Except for HCRTR2 of fishes and birds who lost one flanking gene, all 11 flanking genes were detected in other vertebrates. As for HCRTR1, mammals kept 6 flanking genes and 4 of them changed gene direction compared to HCRTR2. Moreover, what was interesting was that HCRTR1 of fishes had better microsynteny with HCRTR2 rather than HCRTR1 of mammals and HCRTR2 of vase tunicate had no flanking genes in common with HCRTR1 and had one common gene with HCRTR2 of vertebrates.

### 3.4. Protein Structure Domains Analysis

**Figure 4:**
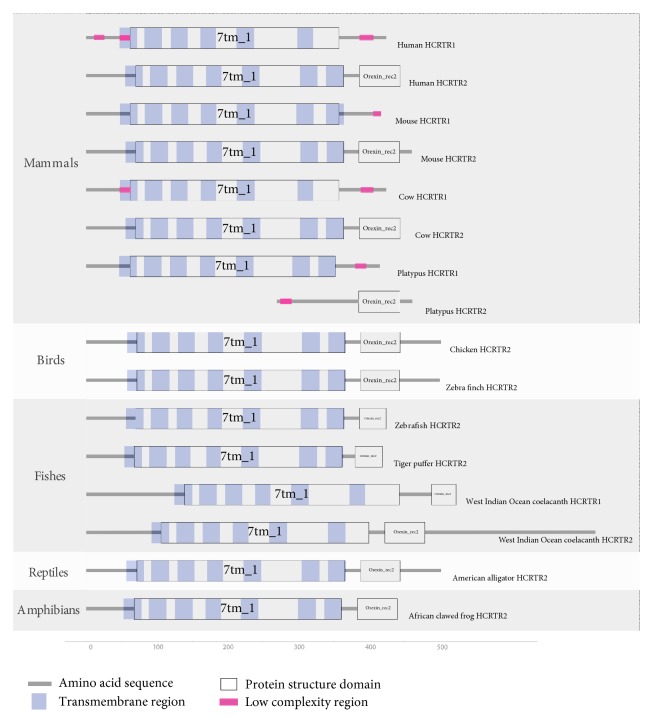
Protein structure domains. We predict protein structures of HCRTR gene family in 109 species. The diagram of 11 representative species is shown. The symbols (keys) stand for different structures.

The diagrams were shown in Figure 4 which illustrated protein structure domains of HCRTRs in 12 representative species. Hcrtr1 contained one protein structure domain (7tm_1) at an average length of 296.2 amino acids and hcrtr2 consisted of two protein structure domains. One domain (7tm_1, average 293.3-amino-acid long) was highly conserved with 7tm_1 in hcrtr1, and the other domain (Orexin_rec2) was specific to hcrtr2 which had 53.4 amino acids on average.

7tm_1 was composed of several transmembrane structures and highly conserved. However, Orexin_rec2 varied among species. It consisted of about 58 amino acids in mammal lineages and about 56 amino acids in birds, amphibians, and reptiles. However, as for fishes, Orexin_rec2 was shorter with only 39 amino acids.

In addition, West Indian Ocean coelacanth, an ancient fish, possessed both hctrt1 and hcrtr2 and hcrtr1 of West Indian Ocean coelacanth had both 7tm_1 and Orexin_rec2.

**Figure 5:**
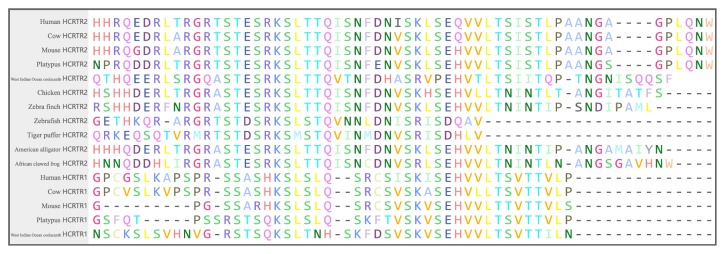
Alignment of corresponding sequences of Orexin_rec2 structure domain. We align the amino acid sequences of HCRTRs of 11 species with MEGE7. Different amino acids are marked in different colors.

In conclusion, differences primarily consisted in the Orexin_rec2. In Figure 5, we compared corresponding amino acid sequences and could see hcrtr1 lost more amino acids than hcrtr2. Hcrtr1 of West Indian Ocean coelacanth lost the least amount of amino acids and it was the only species whose hcrtr1 predicted Orexin_rec2. It suggested that the loss of Orexin_rec2 in hcrtr1 was not owing to the deletion of the whole functional segment but multiple signal amino acid sites. Moreover, low complexity regions (LCRs) of hcrtr1 and hcrtr2 were different in amount. In Figure 4 HCRTR1 of mammals had more LCRs.

### 3.5. Selective Pressure Analysis

Under the optimal model, results (Table 1) indicated hcrtrs were highly conserved in the evolution process. There were lots of negative selection sites detected in all the groups at 0.1 significant level. Only the 143rd site of 7_tm1 structure domain of hcrtr1 among mammals was proved to be undergoing positive selection. And no positive selection sites were found in other two groups of hcrtr2.

## 4. Discussions

To investigate the evolution process of HCRTR gene family, we took advantage of published nucleotide and amino acid sequences, identified or predicted, which were recorded in Additional File 1. HCRTR2 of vase tunicate was the most ancient hypocretin receptor in our study, which revealed that hypocretin receptor had already formed in at least part of Urochordata. HCRTR2 existed in all the vertebrates while HCRTR1 was specific to mammal. This was consistent with previous studies [31]. But 3 HCRTR1-likes of fishes suggested that HCRTR gene family might be larger than anticipated, at least in fishes. The diversity of gene family resulted from evolution.

**Figure 6:**
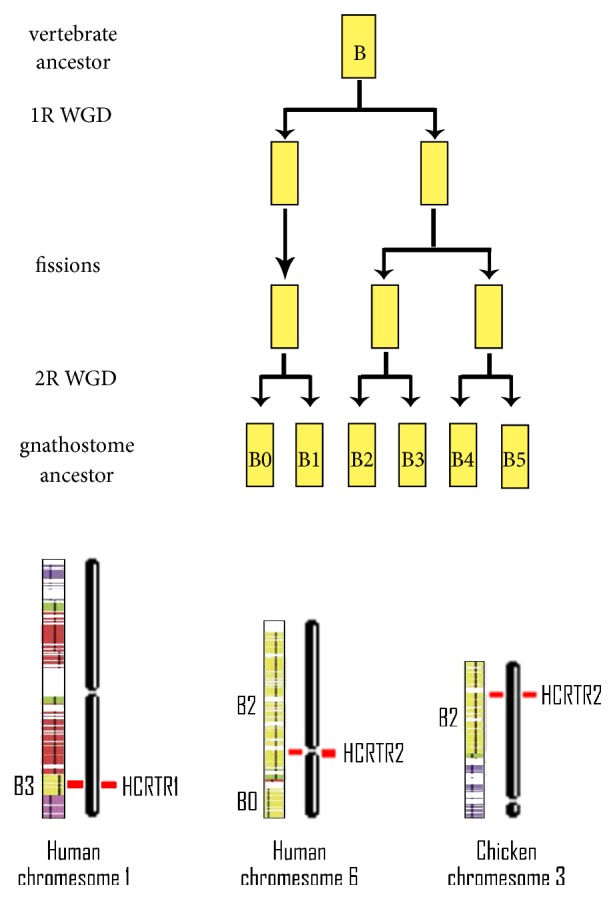
Different regions where HCRTRs are located. The figure illustrates how six vertebrate chromosomes regions are derived from proto-chromosome B. HCRTR1 of human is located roughly in B3 region of chromosome 1 and HCRTR2 is located in B2 region of chromosome 6. HCRTR2 of Chicken is in B2 region of chromosome 3. B2 and B3 regions originated from the second round of WGD.

The phylogenetic trees illustrated that HCRTR1 and HCRTR2 diverged early. And according to gene microsynteny analysis, it could be seen that homology is high between two segments where HCRTR1 and HCRTR2 were located. Gene duplication event had been considered as a reason for new gene. Orthologs were separated by speciation, yet paralogs were generated by gene or genome duplication event [45]. Duplication event was important to gene family expansion. In early vertebrates, the dynamic genome reorganization model [46] indicated that the vertebrate chromosomes were derived from 10 proto-chromosomes through several rounds of whole genome duplications (WGDs, 2 rounds in vertebrates and 3 rounds in fishes) and multiple fissions and fusions. The model divided vertebrate chromosomes into many regions and HCRTR1 of human was located roughly in B3 region of chromosome 1 and HCRTR2 was located in B2 region of chromosome 6. HCRTR2 of Chicken was in B2 region of chromosome 3. Proto-chromosome B was the common ancestor of chromosomes 1 and 6 of human and chromosome 3 of chicken, and B2/B3 regions originated from the second round of WGD (Figure 6). HCRTR gene family was likely to originate in the second round of WGD.

As for the HCRTR1 of fishes, it was different from HCRTR1 of mammals and HCRTR2 of vertebrates according to phylogenetic trees. Moreover, HCRTR1 of fishes had better microsynteny with HCRTR2 rather than HCRTR1 of mammals, which meant that HCRTR1 of fishes and mammals probably did not originate from the same duplication event. The origin of HCRTR1 of fishes might be closely related to the third round of WGD [47] that was specific to the ancestor of fishes. The extra independent genome duplication events had been confirmed in Chinese paddlefish [48] and fishes in the family Salmonidae [49]. Incomplete HCRTR1-like segment was also detected in Atlantic salmon (Salmonidae) in our study.

New genes produced by gene duplication events would not be subject to selective pressure. The structure of HCRTR1 of West Indian Ocean coelacanth suggested that early HCRTR1 might have both 7tm_1 and Orexin_rec2 and then the structure of Orexin_rec2 was destroyed because of relaxed selective pressure. HCRTR2, which HCRTR1 was derived from, was more ancient. It was also supported by gene microsynteny analysis. HCRTR2 of vertebrates had one common flanking gene with HCRTR2 of vase tunicate but HCRTR1 did not. In addition, studies showed that new receptors always acquired ligand binding ability in a new way. Once they acquired this ability, it could gradually develop into specific binding ability [50]. LCRs were reported to be important for the ability of ligand binding [51] and LCRs of hcrtr1 possibly contributed to the specific binding ability, which supported our point as well.

As a vital neuropeptide, it had been well studied that hcrtr1 was a specific receptor to hcrta while hcrtr2 was a nonselective receptor [52]. Due to the structures of hcrtr1 and hcrtr2, we speculated that 7tm_1 mainly responded to hcrta and Orexin_rec2 responded to hcrtb. The remaining structure of Orexin_rec2 of hcrtr1 might account for hcrtr1's inefficient activation by hcrtb [53]. Some held the view that mutations in genes generated by duplication events would not do harms to organisms but actually help them adapt to the environment, such as antifreeze proteins in Antarctic notothenioid fish [54]. HCRTR gene family was highly conserved among species. Selection pressure analysis showed that the negative selection was the predominant evolutionary force acting on the HCRTR gene family, and only one codon was found to be subjected to positive selection in HCRTR1 of mammals. The appearance of HCRTR1 might promise more advanced functions in mammals and fishes.

## 5. Conclusions

The second round of whole genome duplication in early vertebrate ancestry and the independent round in fish ancestry may contribute to the diversity of HCRTR gene family. HCRTR1 of fishes and mammals is not the same receptor, which means that there are three members in the family. HCRTR2 is proved to be the most ancient one in HCRTR gene family. After duplication events, the structure of HCRTR1 diverged from HCRTR2 owing to relaxed selective pressure. Negative selection is the predominant evolutionary force acting on the HCRTR gene family but HCRTR1 of mammals is found to be subjected to positive selection.

## Figures and Tables

**Table 1:** Selective sites in each group.

		**Negative selection**	**Positive selection**				
**Method**	Group	Number	Number	codon	dN-dS	Normalized dN-dS	p-value
**SLAC**	1	123	1	143rd	1.77	1.33	0.084
	2	268	0	-	-	-	-
	3	44	0	-	-	-	-
**FEL**	1	208	1	143rd	0.79	0.59	0.062
	2	269	0	-	-	-	-
	3	48	0	-	-	-	-

## Supplementary Materials

Supplementary Materials The additional file records the information of species and sequences we used in phylogenetic analysis. LPSD1, LPSD2, and LAA mean location of protein structure domain 1 (7tm_1), location of protein structure domain 2 (Orexin_rec2), and length of amino acids, respectively. The highlighted items are parts of the 38 representative species used for Blastn and some species are not listed in the table: black-capped squirrel monkey (XM_010337142.1), Damaraland mole-rat (XM_010628936.1), dromedary (XM_010995387.1), African bush elephant (XM_010587000.1), killer whale (XM_012532256.1), large flying fox (XM_011375020.1), Chinese alligator (XM_006035463.1), and western clawed frog (XM_002934850.3).
